# Vaccine breakthrough infections with SARS-CoV-2 Alpha mirror mutations in Delta Plus, Iota, and Omicron

**DOI:** 10.1172/JCI157700

**Published:** 2022-05-02

**Authors:** Brenda Martínez-González, Lucía Vázquez-Sirvent, María E. Soria, Pablo Mínguez, Llanos Salar-Vidal, Carlos García-Crespo, Isabel Gallego, Ana I. de Ávila, Carlos Llorens, Beatriz Soriano, Ricardo Ramos-Ruiz, Jaime Esteban, Ricardo Fernandez-Roblas, Ignacio Gadea, Carmen Ayuso, Javier Ruíz-Hornillos, Concepción Pérez-Jorge, Esteban Domingo, Celia Perales

**Affiliations:** 1 Department of Clinical Microbiology, Instituto de Investigación Sanitaria-Fundación Jiménez Díaz University Hospital, Universidad Autónoma de Madrid (IIS-FJD, UAM), Madrid, Spain.; 2 Centro de Biología Molecular “Severo Ochoa” (CBMSO), Consejo Superior de Investigaciones Científicas (CSIC-UAM), Campus de Cantoblanco, Madrid, Spain.; 3 Department of Genetics and Genomics, IIS-FJD, UAM, Madrid, Spain.; 4 Centre for Biomedical Network Research on Rare Diseases (CIBERER), Instituto de Salud Carlos III, Madrid, Spain.; 5 Bioinformatics Unit, IIS-FJD, UAM, Madrid, Spain.; 6 Centro de Investigación Biomédica en Red de Enfermedades Hepáticas y Digestivas (CIBERehd), Instituto de Salud Carlos III, Madrid, Spain.; 7 Biotechvana, “Scientific Park,” Universidad de Valencia, Valencia, Spain.; 8 Unidad de Genómica, “Scientific Park of Madrid,” Campus de Cantoblanco, Madrid, Spain.; 9 Allergy Unit, Hospital Infanta Elena, Valdemoro, Madrid, Spain.; 10 IIS-FJD, UAM, Madrid, Spain.; 11 Faculty of Medicine, Universidad Francisco de Vitoria, Madrid, Spain.; 12 Department of Molecular and Cell Biology, Centro Nacional de Biotecnología (CNB), CSIC, Campus de Cantoblanco, Madrid, Spain.

**Keywords:** COVID-19, Virology, Molecular biology

## Abstract

Replication of SARS-CoV-2 in the human population is defined by distributions of mutants that are present at different frequencies within the infected host and can be detected by ultra-deep sequencing techniques. In this study, we examined the SARS-CoV-2 mutant spectra of amplicons from the spike-coding (S-coding) region of 5 nasopharyngeal isolates derived from patients with vaccine breakthrough. Interestingly, all patients became infected with the Alpha variant, but amino acid substitutions that correspond to the Delta Plus, Iota, and Omicron variants were present in the mutant spectra of the resident virus. Deep sequencing analysis of SARS-CoV-2 from patients with vaccine breakthrough revealed a rich reservoir of mutant types and may also identify tolerated substitutions that can be represented in epidemiologically dominant variants.

## Introduction

SARS-CoV-2 continues its diversification worldwide, and a new variant termed Omicron (B.1.1.529), carrying a large number of mutations, was recently described in South Africa and classified as a potential variant of concern (VOC) by the WHO [https://www.who.int/news/item/26-11-2021-classification-of-omicron-(B.1.1.529)-sars-cov-2-variant-of-concern]. As compared with other VOCs, current evidence suggests an increased risk of reinfection with this variant.

It has been reported that distribution of mutants are found during SARS-CoV-2 replication in infected hosts (1–3), as was also previously described for other coronaviruses (4, 5) and in general for RNA viruses. This implies that a consensus sequence of an isolate determined for diagnostic purposes in reality hides a mixture of different variants present in different proportions within the same population (6).

Despite vaccination being highly effective in preventing severe COVID-19, vaccine breakthrough infections have been observed (7, 8). Little is known about the composition of the mutant spectra of SARS-CoV-2 that infect fully vaccinated individuals. This raises the question of whether a vaccine failure could be associated with an ensemble of variant genomes that can facilitate replication in the face of an effective anti–SARS-CoV-2 immune response (9, 10). Here, we show that the virus replicating in vaccinated individuals who developed COVID-19 as a consequence of infection with the Alpha variant included signature mutations of Delta Plus, Iota, and Omicron SARS-CoV-2.

## Results and Discussion

**Figure 1:**
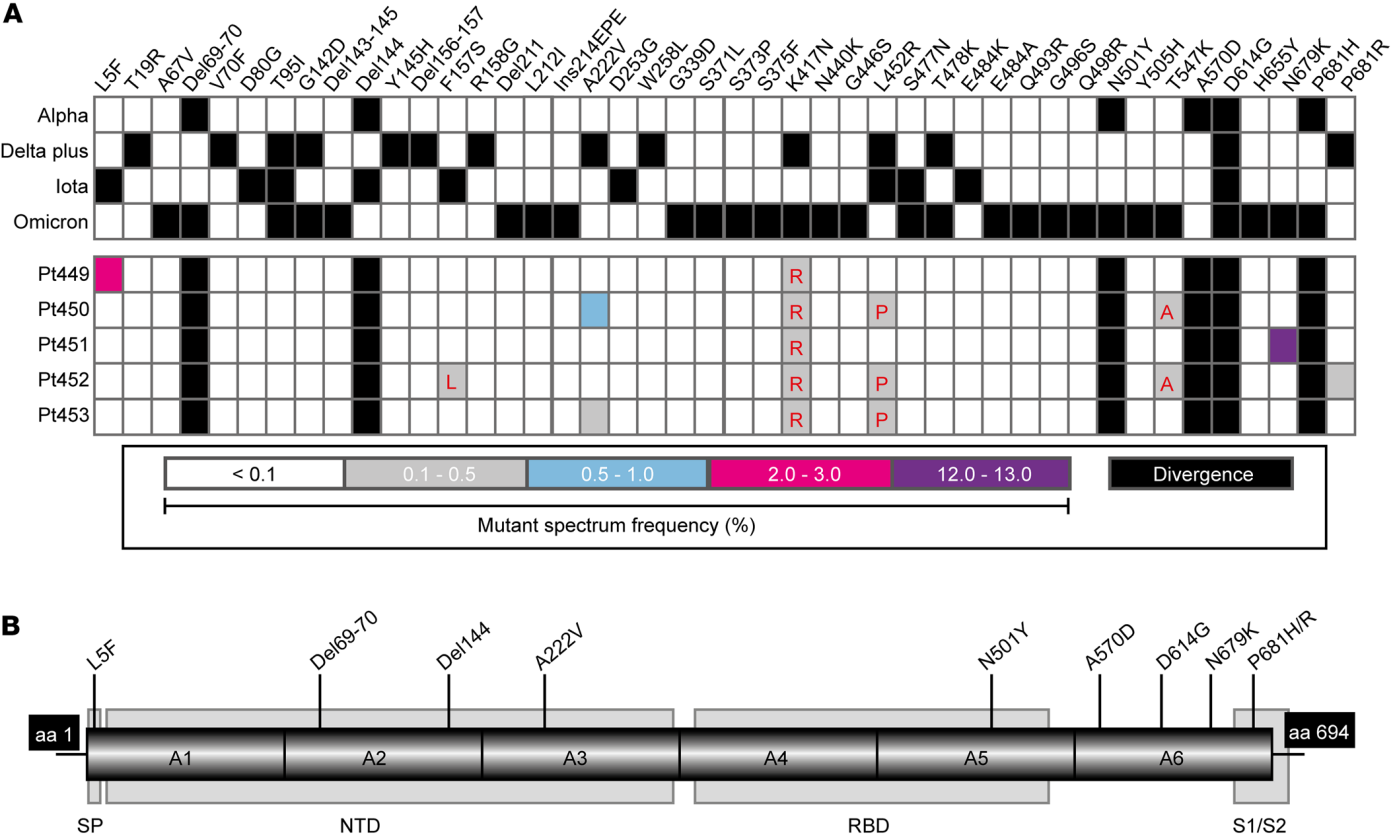
(A) Heatmaps of mutations that define the SARS-CoV-2 variants Alpha, Delta Plus, and Iota as described by the WHO and Omicron as described by Sun et al. (15) (top heatmap) and the corresponding mutations detected in our cohort (*n =* 5) (bottom heatmap). Amino acid substitutions are displayed in the top row, and their presence in the variants Alpha, Delta Plus, Iota, and Omicron is indicated by a black square. The frequency of the substitutions within the mutant spectrum of each sample (with the patient identification code on the left of each row) is color coded, as shown in the bottom box. Substituted amino acids that map at the same position but that are not identical to the substitutions reported for that position in the Delta Plus, Iota, and Omicron variants are indicated in red in the bottom heatmap (from left to right: F157L, K417R, L452P, and T547A). Mutations and deletions are identified relative to the Wuhan-Hu-1 NCBI reference sequence NC_045512.2. (**B**) Representation of the six S amplicons used to perform UDS analysis, with indication of the relevant protein domains: signal peptide (SP), N-terminal domain (NTD), the receptor binding domain (RBD), and the S1/S2 cleavage site (S1/S2). Flanking black boxes indicate the amino acids (aa) of the S protein covered by the amplicons. Mutations and deletions that coincide with the Alpha, Delta Plus, Iota, and Omicron variants found in our patient cohort are indicated.

We studied 5 patients who had been fully vaccinated (2 doses) with BNT162b2 (Pfizer-BioNTech) and who mounted an effective antiviral response (>2000 AU/mL). They were subsequently infected with SARS-CoV-2 in April 2021 and developed COVID-19 clinical symptoms. Nasopharyngeal swabs were collected between April 6, 2021 and April 14, 2021, a time frame that corresponds to the fourth pandemic wave in Madrid, Spain, associated with the Alpha variant. RNA extracted from the diagnostic samples from these vaccinated and infected patients was used to amplify 6 overlapped amplicons of the genomic region of the spike (S) protein (covering nucleotides 21,424 to 23,666; residue numbering is according to the genomic nucleotide sequence of the Wuhan-Hu-1 isolate, NCBI reference NC_045512.2) that were analyzed by ultra-deep sequencing (UDS), with a cutoff value of 0.1%. Two deletions (Δ69–70 and Δ144) and 4 amino acid substitutions (N501Y, A570D, D614G, and P681H), characteristic of the Alpha variant, were dominant variations (termed “divergence” mutations) relative to the reference sequence (Wuhan-Hu-1 isolate) (Figure 1). Interestingly, in addition to these “divergence” mutations, we also found amino acid substitutions representative of the Delta Plus, Iota, and Omicron variants in the mutant spectra of the 5 patients with vaccine breakthrough. In particular, substitution L5F in patient Pt449, present in the Iota variant; A222V in patients Pt450 and Pt453, present in the Delta Plus variant; N679K in patient Pt451, present in the Omicron variant; and P681R in patient Pt452, present in the Delta Plus variant, were found at frequencies of 2.2%, 0.6%, 0.2%, 12.6%, 0.2%, respectively, within their corresponding mutant spectra (Figure 1). Additionally, previously undescribed amino acid replacements at positions that were also substituted by other amino acids in the Iota (amino acids 157 and 452), Delta Plus (amino acids 417 and 452), and Omicron (amino acids 417 and 547) variants were also present (Figure 1).

As a comparison, we analyzed the mutant spectra of diagnostic samples from 5 unvaccinated patients who were infected with the Alpha variant in Madrid at around the same time (January 16, 2021 to February 13, 2021). These virus samples did not include substitution N679K, which was present at a frequency of 12.64% in 1 of the vaccine breakthrough samples. The remaining substitutions were shared by the 2 groups at similar frequencies, with the exception of L5F, which was present at a frequency of 0.24% in virus from 1 of the unvaccinated patients, and 2.2% in virus from a patient with vaccine breakthrough. These data do not support the finding that the majority of substitutions identified in the mutant spectra of virus from patients with vaccine breakthrough were influenced by the immune pressure exerted by the vaccine. Establishing a possible role of N679K in immune escape will require further studies. Thus, despite the fact that the Omicron variant was first reported to the WHO from South Africa on November 24, 2021, the SARS-CoV-2 mutant spectra from an infected patient with vaccine breakthrough from the fourth wave in Madrid already included Omicron-associated mutations.

The presence in the mutant spectra of isolates assigned to the Alpha variant of minority mutations that were dominant in Delta Plus, Iota, and Omicron variants reflects complex intra-host SARS-CoV-2 dynamics, with variants that incorporate tolerated mutations. The variants are present at different frequencies, now amenable to scrutiny by deep-sequencing that can attain cutoff detection levels of 0.1% with the number of clean reads produced (see Methods). Replacements of some minority mutant subpopulations by others are continuously produced, and frequency variations depend on selective pressures applied to the viral population. Mutant spectra may be predictors of the mutation repertoires with the potential to become dominant at the epidemiological level.

The total number of mutations identified in the S-coding region of the SARS-CoV-2 from the 5 patients analyzed amounted to a maximum mutation frequency of 4.09 × 10^–5^ mutations per nucleotide, in line with typical values for RNA viruses in general. This mutational level in nasopharyngeal diagnostic samples suggests the presence of abundant mutation reservoirs. Mutations need not be directly beneficial but may become so in another environment, in a different individual genome sequence, or under another viral population context (intra-mutant spectrum interaction set; ref. 11). Yet another implication is that successive COVID-19 waves that are associated with variants with a defined name cannot be regarded as compartmentalized entities. The mutant spectra of epidemiologically relevant SARS-CoV-2 isolates can be permeated by genomes with minority mutations with past or future prominence.

## Methods

### Patient cohort and stratification.

The virus samples were collected during the fourth COVID-19 outbreak in Spain between April 6, 2021 and April 14, 2021. The study cohort included 5 patients diagnosed as positive for SARS-CoV-2 at the Hospital Universitario Rey Juan Carlos (Móstoles, Madrid, Spain) in April 2021. All patients had been fully vaccinated (2 doses) with BNT162b2 (Pfizer-BioNTech). The patients were considered fully vaccinated, since the second dose of BNT162b2 was administered at least 14 days before they were found to be positive using the standard PCR test for SARS-CoV-2. All patients were confirmed to be positive for SARS-CoV-2, with a Ct between 19 and 30. Data collected included patient demographics, risk factors for SARS-CoV-2 disease, and clinical information related to the time of SARS-CoV-2 diagnosis (Supplemental Table 1; supplemental material available online with this article; https://doi.org/10.1172/JCI157700DS1). The patients were not immunocompromised.

### Oligonucleotide design.

The oligonucleotide primers used for viral RNA amplifications and nucleotide sequencing were designed on the basis of a total of 663 SARS-CoV-2 sequences from the NCBI’s SARS-CoV-2 database (https://www.ncbi.nlm.nih.gov/genbank/sars-cov-2-seqs/). The sequences were retrieved and aligned to the Wuhan-Hu-1 NCBI reference sequence NC_045512.2 (12). The sequences used to design the oligonucleotides are described in Supplemental Table 2. Six pairs of oligonucleotides were used for amplification and sequencing of the overlapping amplicons corresponding to the end of the ORF1b genomic coding region and the genomic region of the S protein (nucleotides 21,424 to 23,666; residue numbering is according to reference sequence NC_045512.2). The nucleotides analyzed encode amino acids 2,661 to 2,6986 of ORF1b and amino acids 1 to 694 of the S protein (Supplemental Table 3).

### RNA extraction and viral RNA amplification of SARS-CoV-2 from infected patients.

SARS-CoV-2 RNA from vaccinated patients was extracted and amplified from 140 μL medium containing nasopharyngeal swab samples using the QIAamp Viral RNA Mini Kit 250 (QIAGEN) according to the manufacturer’s instructions. Amplifications were performed using 5 μL purified RNA solution mixed with 10 μL 5× buffer and 2 μL forward and 2 μL reverse PCR primers (50 ng/μL), and 1 μL polymerase for each amplicon using the Transcriptor One Step RT-PCR Kit (Roche Applied Science). The reverse transcription PCR (RT-PCR) parameters were as follows: 50°C for 30 minutes for the reverse transcription, an initial denaturing step at 94°C for 7 minutes, followed by 45 cycles of a denaturing step at 94°C for 10 seconds, an annealing step at 46°C–48°C for 30 seconds, an extension step at 68°C for 40 seconds, and then a final extension at 68°C for 7 minutes. Amplifications in the absence of RNA were performed in parallel as negative controls. No amplification was observed in any of the negative control runs, and no Iota, Delta Plus, or Omicron SARS-CoV-2 variants were epidemiologically relevant in Spain prior to or during April 2021. The amplification products were analyzed by 2% agarose gel electrophoresis, including the Gene Ruler 1 Kb Plus DNA Ladder (Thermo Fisher Scientific) as the molar mass standard. PCR products were purified using the QIAquick Gel Extraction Kit (QIAGEN), quantified using the Qubit dsDNA Assay Kit (Thermo Fisher Scientific), and, finally, tested for quality (TapeStation System, Agilent Technologies) prior to nucleotide sequencing using the Illumina MiSeq platform.

### UDS of SARS-CoV-2 from infected patients.

To obtain DNA pools, PCR products were adjusted to 4 × 10^9^ molecules/μL and were purified using Kapa Pure Beads (Kapabiosystems, Roche). Pools quantifications were performed using Qubit as previously described and then adjusted to 1.5 ng/μL. DNA pools were processed using the DNA library preparation kit Kapa Hyper Prep (Roche), during which each pool was indexed using the SeqCap Adapter Kit A/B (Nimblegen; 24 Index). Final DNA pools were quantified using the LightCycler 480 (Roche) and sequenced using the Illumina MiSeq sequencing platform with the MiSeq Reagent kit, version 3 (2 × 300 bp mode with the 600 cycle kit).

### Bioinformatics analyses.

Basal error, recombination frequency, and the reproducibility of results were previously performed (13). Given the sequence coverage with 37,311 to 197,230 clean reads per amplicon and patient (Table 1), the mutations considered for the analysis were those with a frequency above a 0.1% cutoff value (Supplemental Table 4). For characterization of the SARS-CoV-2 mutant spectra, the Fastq data were analyzed using the SeekDeep pipeline (14) with the following options: --extraExtractorCmds=-- checkRevComplementForPrimers –primerNumOfMismatches 3” “—extraProcessClusterCmds=--fracCutOff 0.001 –rescueExcludedOneOffLowFreqHaplotypes.”

### Data availability.

Fastq files of the SARS-CoV-2 samples included in the patient cohort are available in the European Nucleotide Archive (ENA) (project ID: PRJEB49400).

### Study approval.

This study was approved by the ethics committee and the IRB of the Fundación Jiménez Díaz (FJD) Hospital (no. PIC-087-20-FJD).

## Author contributions

CP, ED, and CPJ conceived and designed the study. BMG, MES, and LVS performed the experiments. PM, CL, BS, and RRR implemented the computational methods. CPJ, LSV, JE, RFR, and I Gadea provided viral samples and Ct values. LSV, CA, and JRH provided clinical data. CGC, I Gallego, ED, and AIDA analyzed the data. All authors contributed to the writing of the manuscript, and all authors read and approved the final draft.

## Supplementary Material

Supplemental data

## Figures and Tables

**Table 1:**
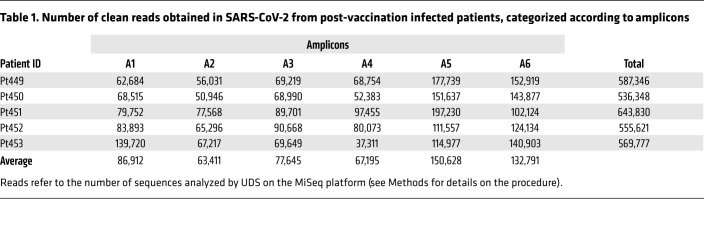
Number of clean reads obtained in SARS-CoV-2 from post-vaccination infected patients, categorized according to amplicons

## Version 1 03/08/2022

In-Press Preview

## Version 2 05/02/2022

Electronic publication
