# Supplementary material for: Vaccine breakthrough infections with SARS-CoV-2 Alpha mirror mutations in Delta Plus, Iota, and Omicron
Source: J Clin Invest. 2022 May 2;132(9):e157700. doi: 10.1172/JCI157700 (PMC9057617; doi:10.1172/JCI157700)
Supplement: Supplemental data [file jci-132-157700-s220.pdf]

Supplementary material for:

Vaccine-breakthrough infections with SARS-CoV-2 Alpha mirror mutations in Delta Plus, Iota and Omicron

Brenda Martínez-González[1], Lucía Vázquez-Sirvent[1], María Eugenia Soria[1,2], Pablo Mínguez[3,4,5], Llanos Salar-Vidal[1], Carlos García-Crespo[2], Isabel Gallego[2,6], Ana Isabel de Ávila[2], Carlos Llorens[7], Beatriz Soriano[7], Ricardo Ramos-Ruiz[8], Jaime Esteban[1], Ricardo Fernandez-Roblas[1], Ignacio Gadea[1], Carmen Ayuso[3,4], Javier Ruíz-Hornillos[9,10,11], Concepción Pérez-Jorge[1], Esteban Domingo[2,6] and Celia Perales[1,6,12*]

[1]Department of Clinical Microbiology, Instituto de Investigación Sanitaria-Fundación Jiménez Díaz University Hospital, Universidad Autónoma de Madrid (IIS-FJD, UAM) Av. Reyes Católicos 2, 28040 Madrid, Spain, [2]Centro de Biología Molecular "Severo Ochoa" (CSIC-UAM), Consejo Superior de Investigaciones Científicas (CSIC), Campus de Cantoblanco, 28049 Madrid, Spain, [3]Department of Genetics & Genomics, Instituto de Investigación Sanitaria-Fundación Jiménez Díaz University Hospital, Universidad Autónoma de Madrid (IIS-FJD, UAM), Av. Reyes Católicos 2, 28040 Madrid, Spain, [4]Centre for Biomedical Network Research on Rare Diseases (CIBERER), Instituto de Salud Carlos III, 28029 Madrid, Spain, [5]Bioinformatics Unit, Instituto de Investigación Sanitaria-Fundación Jiménez Díaz University Hospital, Universidad Autónoma de Madrid (IIS-FJD, UAM), Madrid 28040, Spain, [6]Centro de Investigación Biomédica en Red de Enfermedades Hepáticas y Digestivas (CIBERehd), Instituto de Salud Carlos III, 28029 Madrid, Spain, [7]Biotechvana, "Scientific Park", Universidad de Valencia, 46980 Valencia, Spain, [8]Unidad de Genómica, "Scientific Park of Madrid", Campus de Cantoblanco, 28049 Madrid, Spain, [9]Allergy Unit, Hospital Infanta Elena, Valdemoro, Madrid, Spain; [10]Instituto de Investigación Sanitaria-Fundación Jiménez Díaz University Hospital, Universidad Autónoma de Madrid (IIS-FJD, UAM), Av. Reyes Católicos 2, 28040 Madrid, Spain; [11]Faculty of Medicine, Universidad Francisco de Vitoria, Madrid, Spain, [12]Department of Molecular and Cell Biology, Centro Nacional de Biotecnología (CNB-CSIC), Consejo Superior de Investigaciones Científicas (CSIC), Campus de Cantoblanco, 28049 Madrid, Spain

*Address correspondence to: Celia Perales, Centro Nacional de Biotecnología (CNB-CSIC), Consejo Superior de Investigaciones Científicas (CSIC), C/ Darwin, 3, Campus de Cantoblanco, 28049 Madrid, Spain. Phone: +34 91 196 4541; Email: cperales@cbm.csic.es; celia.perales@cnb.csic.es

Table S1. Demographic data and preexisting comorbidities in five SARS-CoV-2 infected patients after vaccination.

Characteristics	TOTAL (n=5)
**Age mean ± SD, years**	61.4 ± 25
**Male (%)**	2 (40%)
**Female (%)**	3 (60%)
**Hypertension (%)**	3 (60%)
**Cardiac disease (%)**	2 (40%)
**Diabetes (%)**	1 (20%)
**Dyslipidemia (%)**	2 (40%)
**Pulmonary pathology (%)**	1 (20%)
**Smoker (%)**	0
**Hospitalization (%)**	1 (20%)
**Antibodies after vaccination (%)** [a]	4 (80%)
**Survival 90 days after diagnosis (%)**	5 (100%)
**Ct (cycle threshold, which is inversely correlated with viral RNA level) ± SD**	22.0 ± 4.2

[a] Data available for four patients. All patients in whom the level of antibodies was determined after vaccination, the result was greater than 2,000 (>50 it is considered positive).

Table S2. Reference accession numbers of sequences retrieved from the National Center for Biotechnology Information (NCBI) database to design specific oligonucleotides.

NC_045512.2; NC_045512; MN908947.3; MT510727.1; MT510728.1; MT509958.1; MT509959.1; MT509649.1; MT509650.1; MT509651.1; MT509656.1; MT509657.1; MT509658.1; MT509659.1; MT509494.1; MT509495.1; MT509496.1; MT509497.1; MT509498.1; MT509499.1; MT509500.1; MT509501.1; MT509502.1; MT509503.1; MT509504.1; MT509505.1; MT509506.1; MT509507.1; MT509508.1; MT509509.1; MT509510.1; MT509511.1; MT509512.1; MT500122.1; MT502774.1; MT502900.1; MT502901.1; MT502902.1; MT502903.1; MT502904.1; MT502905.1; MT502906.1; MT502907.1; MT502908.1; MT502909.1; MT502910.1; MT502911.1; MT502912.1; MT502913.1; MT502914.1; MT502915.1; MT502916.1; MT502917.1; MT502918.1; MT502919.1; MT502920.1; MT502921.1; MT502922.1; MT502923.1; MT502924.1; MT502925.1; MT502926.1; MT502927.1; MT502928.1; MT502929.1; MT502930.1; MT502931.1; MT502932.1; MT502933.1; MT502934.1; MT502935.1; MT502936.1; MT502937.1; MT502938.1; MT502939.1; MT502940.1; MT502941.1; MT502942.1; MT502943.1; MT502944.1; MT502945.1; MT502946.1; MT502947.1; MT502948.1; MT502949.1; MT502950.1; MT502951.1; MT502952.1; MT502953.1; MT502954.1; MT502955.1; MT502956.1; MT502957.1; MT502958.1; MT502959.1; MT502960.1; MT502961.1; MT502962.1; MT502963.1; MT502964.1; MT502965.1; MT502966.1; MT502967.1; MT502968.1; MT502969.1; MT502970.1; MT502971.1; MT502972.1; MT502973.1; MT502974.1; MT502975.1; MT502976.1; MT502977.1; MT502978.1; MT502979.1; MT502980.1; MT502981.1; MT502982.1; MT502983.1; MT502984.1; MT502985.1; MT502986.1; MT502987.1; MT502988.1; MT502989.1; MT502990.1; MT502991.1; MT502992.1; MT502993.1; MT502994.1; MT502995.1; MT502996.1; MT502997.1; MT502998.1; MT502999.1; MT503000.1; MT503001.1; MT503002.1; MT503003.1; MT503004.1; MT503005.1; MT503006.1; MT503007.1; MT503008.1; MT503009.1; MT503010.1; MT503011.1; MT503012.1; MT503013.1; MT503014.1; MT503015.1; MT503016.1; MT503017.1; MT503018.1; MT503019.1; MT503020.1; MT503021.1; MT503022.1; MT503023.1; MT503024.1; MT503025.1; MT503026.1; MT503027.1; MT503028.1; MT503029.1; MT503030.1; MT503031.1; MT503032.1; MT503033.1; MT503034.1; MT503035.1; MT503036.1; MT503037.1; MT503038.1; MT503039.1; MT503040.1; MT503041.1; MT503042.1; MT503043.1; MT503044.1; MT503045.1; MT503046.1; MT503047.1; MT503048.1; MT503049.1; MT503050.1; MT503051.1; MT503052.1; MT503053.1; MT503054.1; MT503055.1; MT503056.1; MT503057.1; MT503058.1; MT447174.1; MT447175.1; MT447176.1; MT447177.1; MT447188.1; MT447189.1; MT439595.1; MT439596.1; MT439597.1; MT434757.1; MT434758.1; MT434759.1; MT434760.1; MT435079.1; MT435080.1; MT435081.1; MT435082.1; MT435083.1; MT435084.1; MT435085.1; MT435086.1; MT365028.1; MT365029.1; MT365030.1; MT365031.1; MT365032.1; MT428551.1; MT428552.1; MT428553.1; MT428554.1; MT429168.1; MT215193.1; MT215194.1; MT215195.1; MT270814.1; MT270815.1; MT276600.1; MT412134.1; MT415320.1; MT415321.1; MT415322.1; MT415323.1; MT415833.1; MT415834.1; MT415835.1; MT415836.1; MT415837.1; MT415838.1; MT415839.1; MT415366.1; MT415367.1; MT415368.1; MT415369.1; MT415370.1; MT415371.1; MT415372.1; MT415373.1; MT415374.1; MT415375.1; MT415376.1; MT415377.1; MT415840.1; MT415841.1; MT415842.1; MT415843.1; MT415844.1; MT415845.1; MT415846.1; MT396241.1; MT396242.1; MT396243.1; MT396244.1; MT396245.1; MT396246.1; MT396247.1; MT396248.1; MT380726.1; MT380727.1; MT186683.1; MT374101.1; MT374102.1; MT374103.1; MT374104.1; MT374105.1; MT374106.1; MT374107.1; MT374108.1; MT374109.1; MT374110.1; MT374111.1; MT374112.1; MT374113.1; MT374114.1; MT374115.1; MT374116.1; MT370516.1; MT370517.1; MT370518.1; MT371047.1; MT371048.1; MT371049.1; MT371050.1; MT372480.1; MT372481.1; MT372482.1; MT372483.1; LC542976.1; LC542809.1; MT114412.1; MT114413.1; MT114414.1; MT114415.1; MT114416.1; MT114417.1; MT114418.1; MT114419.1; MT230904.1; MT358637.1; MT327745.1; MT039874.1; MT320891.2; MT304474.1; MT304475.1; MT304476.1; MT281577.1; MT291831.1; MT291832.1; MT291833.1; MT291834.1; MT291835.2; MT291836.1; MT281530.2; MT291827.1; MT291828.1; MT291826.1;

MT291829.1; MT291830.1; MT262993.1; MT259226.1; MT259227.1; MT259228.1; MT259229.1; MT259230.1; MT259231.1; LC534418.1; MT253696.1; MT253697.1; MT253698.1; MT253699.1; MT253700.1; MT253701.1; MT253702.1; MT253703.1; MT253704.1; MT253705.1; MT253706.1; MT253707.1; MT253708.1; MT253709.1; MT253710.1; MT240479.1; MT226610.1; MT192772.1; MT192773.1; MT121215.1; LC529905.1; MT192759.1; MT135041.1; MT135042.1; MT135043.1; MT135044.1; MT503059.1; MT503072.1; MT503063.1; MT503068.1; MT503060.1; MT503061.1; MT503062.1; MT503064.1; MT503065.1; MT503066.1; MT503067.1; MT503069.1; MT503070.1; MT503071.1; MT503073.1; MT503074.1; MT503075.1; MT503076.1; MT503077.1; MT503078.1; MT503079.1; MT503080.1; MT503081.1; MT503082.1; MT503083.1; MT503084.1; MT503085.1; MT503097.1; MT503086.1; MT503087.1; MT503089.1; MT503090.1; MT503091.1; MT503092.1; MT503093.1; MT503094.1; MT503095.1; MT481895.1; MT481896.1; MT481897.1; MT481898.1; MT481899.1; MT481900.1; MT481901.1; MT481902.1; MT481903.1; MT481904.1; MT481905.1; MT481906.1; MT481907.1; MT481908.1; MT481909.1; MT496972.1; MT496973.1; MT496974.1; MT496975.1; MT496976.1; MT496977.1; MT496978.1; MT496979.1; MT496980.1; MT496982.1; MT496983.1; MT496984.1; MT496985.1; MT496986.1; MT496987.1; MT496988.1; MT496989.1; MT496990.1; MT496991.1; MT496992.1; MT496993.1; MT496994.1; MT496995.1; MT496996.1; MT496997.1; MT483553.1; MT483554.1; MT483555.1; MT483556.1; MT483557.1; MT483558.1; MT483559.1; MT483560.1; MT483702.1; MT477885.1; MT496981.1; LC547518.1; LC547519.1; MT503088.1; MT503096.1; LC546038.1; MT503098.1; MT503099.1; LC547520.1; LC547529.1; LC547526.1; LC547525.1; LC547523.1; LC547533.1; LC547527.1; LC547528.1; LC547530.1; LC547531.1; LC547532.1; LC547521.1; LC547522.1; LC547524.1; MT467237.1; MT467238.1; MT467239.1; MT467240.1; MT467241.1; MT467242.1; MT467243.1; MT467244.1; MT467245.1; MT467246.1; MT467247.1; MT467248.1; MT467249.1; MT467250.1; MT467251.1; MT467252.1; MT467253.1; MT467254.1; MT467255.1; MT467256.1; MT467257.1; MT467258.1; MT467259.1; MT467260.1; MT467261.1; MT467262.1; MT467263.1; MT459928.1; MT457402.1; MT457403.1; MT450425.1; MT450426.1; MT450427.1; MT450428.1; MT450429.1; MT450430.1; MT450431.1; MT450432.1; MT450433.1; MT450434.1; MT450435.1; MT450436.1; MT450437.1; MT450438.1; MT451874.1; MT451875.1; MT451876.1; MT451877.1; MT451878.1; MT451879.1; MT451880.1; MT451881.1; MT451882.1; MT451883.1; MT451884.1; MT451885.1; MT451886.1; MT451887.1; MT451888.1; MT451889.1; MT451890.1; MT446312.1; MT447154.1; MT447155.1; MT447156.1; MT447157.1; MT447158.1; MT447159.1; MT447160.1; MT447161.1; MT447162.1; MT447163.1; MT447164.1; MT447165.1; MT447166.1; MT447167.1; MT447168.1; MT447169.1; MT447170.1; MT447171.1; MT447172.1; MT447173.1; MT079851.1; MT476385.1; MT079844.1; MT079845.1; MT079846.1; MT079843.1; MT079847.1; MT079848.1; MT079849.1; MT079850.1; MT079852.1; MT079854.1; MT079853.1; MT350234.1; MT293547.1; MT273658.1; MT232869.1; MT232870.1; MT232871.1; MT232872.1; MT186676.1; MT186677.1; MT186678.1; MT186679.1; MT186680.1; MT186681.1; MT186682.1; MT163714.1; MT163715.1; MT163737.1; MT163738.1; MT012098.1; MT050493.1; MT049951.1; MT039890.1; MT019529.1; MT019530.1; MT019531.1; MT019532.1; MT019533.1; MN996528.1; MN975262.1; MT066175.1; MT066176.1; MT081059.1; MT081060.1; MT081061.1; MT081062.1; MT081063.1; MT081064.1; MT081065.1; MT081066.1; MT081067.1; MT081068.1; MT072688.1; MT066157.1; MT066158.1; MT066159.1; LC523807.1; LC523808.1; LC523809.1; MT042773.1; MT042774.1; MT042775.1; MT042776.1; MT042777.1; MT042778.1; MN938387.1; MN938388.1; MN938389.1; MN938390.1; MN975266.1; MN975267.1; MN975268.1; MN938385.1; MN975263.1; MN975264.1; MN975265.1; MN938386.1; MT039873.1; MN988668.1; MN988669.1; MN970003.1; MN970004.1; LC522350.1; LR757996.1; LR757998.1; MN996527.1; MN996529.1; MN996530.1; MN996531.1; MN938384.1; MT152900.1; MT127113.1; MT127115.1; MT127116.1; MT123291.2; MT123292.2; MT123293.2; MT093631.2; MT276597.1; LR757995.1; LC534419.1; MT123290.1; MT276598.1; MT163712.1; MT127114.1

Table S3. Specific oligonucleotides designed to amplify and sequence the spike-coding region of SARS-CoV-2.

Primer name	Sense	Sequence (5' – 3')	Genome positions[a]	Tm (ºC)
Spk-CoV2-u21424	Fw	GGTACTGCTGTTATGTCTTTAAAA	21424-21447	50.6
Spk-CoV2-d21867	Rv	CTTATTATGTTAGACTTCTCAGTGGA	21867-21842	53.2
Spk-CoV2-u21701	Fw	GTTTTACATTCAACTCAGGACTTGTT	21701-21726	53.2
Spk-CoV2-d22154	Rv	CAATATTCTTAAACACAAATTCCCTAA	22154-22128	50.6
Spk-CoV2-u22088	Fw	CTTATGGACCTTGAAGGAAAACA	22088-22110	51.7
Spk-CoV2-d22536	Rv	GATTCTGTTGGTTGGACTCTA	22536-22516	50.5
Spk-CoV2-u22464	Fw	GTACGTTGAAATCCTTCACTGTA	22464-22486	51.7
Spk-CoV2-d22902	Rv	CCACCAACCTTAGAATCAAGAT	22902-22881	51.1
spk-CoV2-u22853	Fw	GGCTGCGTTATAGCTTGGA	22853-22871	51.1
spk-CoV2-d23288	Rv	CAGCATCAGTAGTGTCAGCA	23288-23269	51.8
spk-CoV2-u23236	Fw	GTTTCTGCCTTTCCAACAATTTG	23236-23258	51.7
spk-CoV2-d23666	Rv	CTGCACCAAGTGACATAGTGT	23666-23646	52.4

[a]The SARS-CoV-2 genome residue numbering according to the NCBI reference sequence (accession number NC_045512.2).

Table S4. Repertoire of substitutions and deletions in the spike-coding region of SARS-CoV-2 (amino acid positions 1–694)[a], analyzed by ultra-deep sequencing (UDS).

**Region**	**Amino Acid substitution**[b]	**PAM250**[c]	**Number of patients**	**Domain**
spike A1	L5F	2	1	Signal peptide
spike A2	F157L	2	1	N-terminal
spike A3	A222V	0	2	N-terminal
spike A4	K417R	3	5	RBD
spike A5	L452P	-3	3	RBD
spike A5	N501Y	-2	5	RBD
spike A5	T547A	1	2	-
spike A6	A570D	1	5	-
spike A6	D614G	1	5	-
spike A6	N679K	1	1	-
spike A6	P681H	0	5	S1/S2
spike A6	P681R	0	1	S1/S2

**Region**	**Affected amino acid positions**[b]	**Amino acid reference sequence**[d]	**Amino acid sequence detected**[h]	**Number of patients**
spike A1/ A2	69-70	AIHVSG	AISG	5
spike A2	144	GVYYH	GVYH	5

[a] The SARS-CoV-2 genome residue numbering according to the NCBI reference sequence (accession number NC 045512.2).

[b] Amino acid residues (single letter code) are numbered from N- to the C-terminus of each region.

[c] PAM250 substitution matrix values are take from Feng and Doolittle (Methods in Enzymology 266: 368-382, 1996): PAM250<0, lower acceptability than expected, meaning a rare replacement; PAM250=0, acceptability as expected; PAM250>0 acceptability higher than expected.

[d] Underline amino acids are the ones affected by the deletion.

[h] Amino acid sequence detected in patients.
